# Comparison of the detection rate of cervical lesion with TruScreen, LBC test and HPV test: A Real-world study based on population screening of cervical cancer in rural areas of China

**DOI:** 10.1371/journal.pone.0233986

**Published:** 2020-07-07

**Authors:** Yu Ma, Jiangli Di, Hui Bi, Qingping Zhao, Tianhua Qin, Wen Xu, Zhaoyang Liu, Nianhua Yi, Jing Zhao, Deping Zhou, Jiancui Chen, Qi Yang

**Affiliations:** 1 National Center for Women and Children’s Health, Chinese Center for Disease Control and Prevention, Beijing, China; 2 Department of Obstetrics and Gynecology, Peking University First Hospital, Beijing, China; 3 Department of Gynecology, Sichuan Provincial Hospital for Women and Children, Chengdu, China; 4 Department of Gynecological Clinic, Xinjiang Uygur Autonomous Region Maternal and Child Health Hospital, Ürümqi, China; 5 Women’s Health Care Department, Haidian Maternal and Child Health Hospital, Beijing, China; 6 Department of Gynecology, Northwest Women’s and Children’s Hospital (Shaanxi Maternal and Child Health Hospital), Xi’an, China; 7 Department of Women’s Health Care, Maternal and Child Health Hospital of Hubei Province, Wuhan, China; 8 Department of Gynecology, Hunan Provincial Maternal and Child Health Hospital, Changsha, China; 9 Cervical Disease Treatment Center, Chongqing Health Center for Women and Children, Chongqing, China; 10 Cervical Disease Diagnosis and Treatment Health Center, Fujian Provincial Maternity and Children’s Hospital, Affiliated Hospital of Fujian Medical University, Fuzhou, China; Ordu University, TURKEY

## Abstract

**Background and objective:**

China carries a heavy burden of cervical cancer and has an alarmingly low cervical cancer screening rate. In order to achieve the goal of cervical cancer elimination, there is an urgent need for suitable methods and strategies in China.

**Materials and methods:**

A total 9972 woman who received cervical cancer screening services of National Cervical Cancer Screening Program in Rural Areas (NCCSPRA) in 8 project counties participated in this study. TruScreen, HPV test and LBC test were performed in all participants. A total of 1945women had one or more than one positive or abnormal screening results of the above three screening tests subsequently received colposcopy. The detection rate of CIN2+ between the three tests were compared.

**Results:**

No matter what kind of screening method is used, the CIN2+ detection rate in the eastern regions was much higher than that in the central and western regions. The total detection rate of CIN2+ in HPV group was highest (0.73%), following in LBC group (0.44%) and TS group (0.31%). There was statistically significant difference in the total detection rate of CIN2+ between TS and HPV groups, LBC and HPV groups, respectively. There was no statistical difference in the total detection rate of CIN2+ between TS and LBC screening groups. Moreover, except for the eastern regions, there was no statistical difference in the detection rate of CIN2+ between TS group and the other two groups in central and western regions.

**Conclusion:**

If it can meet the requirements of the laboratory and personnel, HPV test seems to be the preferred method for cervical cancer screening in rural areas of China. The characteristics of minimal training requirements, simple operation, real-time results obtained without the collection of cervical cell samples and the help of laboratory equipment and cytologists of TS make it ideal for cervical cancer screening in low-resource regions.

## Introduction

Worldwide, cervical cancer affects over half a million women each year, and kills a quarter of a million [1].Without action, deaths from cervical cancer will rise by almost 50% by 2030 [2]. Because of its large population, China carries a heavy burden of cervical cancer. Meanwhile, there are obvious regional differences in the incidence and mortality of cervical cancer in China, and the incidence of and the mortality for cervical cancer has an upward trend since 2000 [3, 4]. In2009,in order to reduce the high cervical cancer burden in rural areas, the Chinese government launched the National Cervical Cancer Screening Program in Rural Areas (NCCSPRA) [5]. However, there were some weaknesses and threats [6]–shortage of cytological reading personnel, limited service capacity of health care institutions in remote and poor areas, large population for screening–to effect implementation of the screening program and cause challenges for a proper population based screening especially in central and western regions [7]. In 2018, WHO made a global call for action towards the elimination of cervical cancer and set a goal of 70% of women screened with a high precision test at 35 and 45 years of age by 2030 [1]. However, at present, there is an alarmingly low cervical cancer screening rate (26.7%) among women aged35–64 years in China [8]. Hence, for China, to achieve the goal of cervical cancer elimination, there is an urgent need to explore and evaluate innovative screening methods to improve the effectiveness of cervical cancer screening.

Artificial intelligence (AI) has developed rapidly in recent years, and it has also had a far-reaching impact on cervical cancer screening. One of the AI techniques that has aroused the interest of health workers in this area is TruScreen (TS) (Polarprobe; Polartechnics, Australia), which uses optical and electrical stimulation to detect carcinogenesis and precancerous lesions in cervical tissue, with the characteristics of being real-time and non-invasive [9]. However, at present, most of the studies on cervical cancer screening by TS have been based on small sample clinical patients, and the evaluation of the screening effect of TS has shown wide differences in different studies. Some studies showed that the effect of TS is consistent with that of cytology, but some studies show that its screening effect is not as good as that of cytology [10–12]. So in the real world, is there any difference in the detection rate of cervical lesion between TS and human papillomavirus (HPV) test and Liquid-based cytology (LBC) test which currently used in the project area of NCCSPRA? Which is suitable for cervical cancer screening at the grass-roots level in China? All these are worthy of our further research on large samples, multi-centers and population-based screening.

Real-world study (RWS) refers to a study that obtains a variety of data in a real clinical, community or family environment to evaluate the real impact [13]. RWS has the following characteristics: wide coverage, usually large sample size; grouping based on the will of participants or clinical choice, not necessarily randomized. The evaluation results of RWS are based on the real environment, and the external authenticity is good and the evidence resources are more abundant [14].The purpose of the real world study is to summarize the diagnosis and treatment data of real clinical patients, so as to make the research results more instructive to the practical work [15]. This study is based on a real-world study of people undergoing cervical cancer screening conducted by grass-roots personnel in the rural cervical cancer screening project in China. The purpose of this study is to provide a scientific basis for policy makers to formulate the cervical cancer screening strategies suitable for the actual situation of our country.

## Materials and methods

### Participants and procedures

This study was conducted from August to December 2018. Considering the influence of economic development and geographical location on the incidence and screening effect of cervical cancer, a multi-stage sampling method was adopted to divide the country into 3 regions (eastern, central and western regions) according to geographical location and economic development level. Two and four NCCSPRA project counties which used LBC test as the screening method in eastern (Haidian district in Beijing, Luoyuan county in Fujian province),central (Zaoyang in Hubei province, Pingjiang county in Hunan province) and western regions (Aletai, in Xinjiang Uygur Autonomous region, Deyang county in Sichuan province, Hechuan county in Chongqing, Hancheng county in Shaanxi province) respectively were selected as the study areas.

A total 10,024 woman who received cervical cancer screening services of NCCSPRA in 8 project counties were recruited in this study. Women with intact cervix, without history of cervical disease, without cervical biopsy, microwave, laser, radiotherapy and chemotherapy treatments within 3 months were included in this study. Women with severe uncontrollable bleeding in the cervix, women who declined to participate, who were lost to follow-up, who suffered from photosensitive diseases or who had been exposed to photosensitive treatments were excluded.

All participants provided their written consent to participate in this study and all were informed about confidentially measures and rights to withdraw. This study was approved by the Ethics Committee of the National Women and Children's Health Center, China CDC (No:FY2018-08).

Using a structured questionnaire, information about demographics and disease history of each woman was obtained before clinical and laboratory examination by trained investigators. All woman received gynecologic pelvic examination and were screened by TS examination, HPV test (DH3, DALTONbio) and LBC test(D.A Diagnostics, Hologic, Kingmed Diagnostics). The order of examination and sampling was TS examination, LBC sampling, HPV sampling and pelvic examination. The women with positive TS or HPV 16/18 infection or abnormal cytology (atypical squamous cells of undetermined significance, ASC-US+) were referred for colposcopy. The women with positive infection of the other 12 high-risk subtypes of HPV (HPV 31,33,35,39,45,51,52,56,58,59,66 and 68) were referred for LBC and TS. The women with abnormal results of colposcopy (low-grade lesions, high-grade lesions or suspected cancer) underwent biopsy. TS examination, LBC sampling, HPV sampling, colposcopy, biopsy, and cytology and histology interpreted were performed by the grass-roots personnel who were involved in the NCCSPRA. The HPV samples were uniformly collected and sent to the testing institutions requested by the research for testing. Colposcopy was performed according to accepted diagnostic standards and national guidelines. Biopsy confirmed CIN2+ was used as clinical end point for the study.

### Statistical analysis

Pearson’s chi-squared test was used to compare differences in detection rate of CIN2+ between different screening methods groups. $Detection\;rate\;of\;\mathrm{CIN}2+=\frac{\mathrm{Number}\;\mathrm{of}\;\mathrm{CIN}2+}{\mathrm{Number}\;\mathrm{of}\;\mathrm{screenig}\;\mathrm{women}}\times 100$ *p<*0.05 indicated a significant difference between groups and *p<*0.017 indicated a significant difference between subgroups.

## Results

A total of 9972 women met the inclusion criteria. Of the 9972 participants, 2484(24.9%) were from the eastern region, 2502 (25.1%) from the central region, and 4986 (50.0%) from the western region. The mean age of the participants was 45.2±5.4 years.

The overall prevalence of positive results by TS, HPV and LBC was12.2%,7.5% and 3.1%, respectively. The proportions of positive results of TS and LBC were highest in the eastern regions(14.4% and 4.8%, respectively). The proportion of positive results of HPV in the western regions was higher than in the other regions (Table 1).

**Table 1:** Positive results of TS, HPV and LBC screening indifferent regions of China, 2018.

Regions	N	TS		HPV		LBC	
		n	%	n	%	n	%
Eastern	2484	358	14.4	192	7.7	120	4.8
Central	2502	319	12.8	160	6.4	64	2.6
Western	4986	544	10.9	400	8.0	121	2.4
Total	9972	1221	12.2	752	7.5	305	3.1

A total of 1945 (19.5%) women had one or more than one positive or abnormal screening results and needed further colposcopy. Among them, 96.8% (1882/1945) received colposcopy. The follow-up rate of colposcopy in the central region was highest (99.8%) (Table 2).

**Table 2:** Situation of follow-up of colposcopy and CIN2+ detection in different regions of China.

Regions	N	Need to receive colposcopy		Actually performed colposcopy	
		n	%	n	%
Eastern	2484	563	22.7	546	97.0
Central	2502	469	18.8	468	99.8
Western	4986	913	18.3	868	95.1
Total	9972	1945	19.5	1882	96.8

The total detection rate of CIN2+ by HPV group was highest (0.73%), followed by LBC (0.44%) and TS (0.31%). There were statistically significant differences in the total detection rate of CIN2+ between TS and HPV groups (χ^2^ = 17.050, *P<*0.001), and LBC and HPV groups (χ^2^ = 7.230, *P* = 0.007). However, there was no statistical difference in the total detection rate of CIN2+ between TS and LBC groups (χ^2^ = 2.262, *P* = 0.133). Only in the eastern region, there was a statistically significant difference in the detection rate of CIN2+ between TS and HPV groups (χ^2^ = 11.363, *P* = 0.001). In the other regions, there was no statistical difference in the detection rate of CIN2+ between different screening methods (Table 3).

**Table 3:** Detection rate of CIN2+ in different screening methods groups in different regions of China.

Screening methods	Eastern	Central	Western	Total
	N(%)	n(%)	n(%)	n(%)
TS	12**(**0.48**)**^a^	5**(**0.20**)**	14**(**0.28**)**	31**(**0.31**)**^a^
HPV	35**(**1.41**)**^a^	12**(**0.48**)**	26**(**0.52**)**	73**(**0.73**)**^a^^b^
LBC	21**(**0.85**)**	7**(**0.28**)**	16**(**0.32**)**	44**(**0.44**)**^b^
χ^2^	11.962	3.260	4.445	18.836
*p*	0.003	0.196	0.108	<0.001

a indicates a statistically significant difference between TS and HPV groups, *p*<0.017

b indicates a statistically significant difference between HPV and LBC groups, *p*<0.017

## Discussion

Some developed countries have significantly reduced the incidence of and mortality from cervical cancer through the implementation of a national organized screening programs [16, 17], however, in some developing countries (e.g. Costa Rica, Colombia and Puerto Rico), the results have not been so positive [18, 19]. When comparing performance of cervical cancer screening programs across different countries, it was clear that the service capacity of health care institutions in different areas, the number of cytological readers, laboratory test conditions and population acceptance and satisfaction with screening methods have an impact on the effectiveness of large-scale population screening [20–22]. Therefore, since the implementation of NCCSPRA in 2009, researchers have been exploring a cervical cancer screening method which can minimize the above impact factors and is suitable for China's rural areas. As the primary screening method recommended by the National Health Commission (NHC) of China, cytological test and HPV test are the main methods used for cervical cancer screening in NCCSPRA [23, 24]. As a new real-time screening method, some studies showed that TS can be another more appropriate screening method in developing countries because of its advantages of non-invasive, instant diagnosis, decreases the need for pathologists, portable, and similar sensitivity and specificity to HPV and LBC tests [25, 26]. However, which of the above methods is more suitable for cervical cancer screening program in rural areas of China, this study based on real world conditions is particularly important. This is the first multicenter, large sample, population-based study, based on a real-world study of people under going cervical cancer screening on TS, HPV, and LBC screening conducted by grass-roots personnel in the rural cervical cancer screening project in China.

The results of this study showed that no matter what kind of screening method is used, the CIN2+ detection rate in the eastern region was much higher than that in the central and western regions. However, the results of *Report of cancer incidence and mortality in different areas of China*, *2014* [27] showed that the incidence of cervical cancer in eastern region**(**world standard population, ASIRW: 9.31/100,000**)**was lower than that in central (ASIRW: 12.25/100,000) and western regions (ASIRW: 10.51/100,000). The results of the national surveys [6, 7] showed that the number and the capacities of medical staff in cytology and pathology in the central and western regions were far less and much lower than those in the eastern regions. This also proves once again that no matter what kind of screening method, personnel ability and service quality are very important factors that affect the effectiveness of detection.

The results of this study showed that the total detection rate of CIN2+ by HPV was highest (0.73%), followed by LBC (0.44%) and TS (0.31%). Moreover, there were statistically significant differences in the total detection rates of CIN2+ between TS and HPV, and LBC and HPV, respectively. Also, HPV test has the following advantages: simple process for collection of specimens, the possibility of self-collected specimens, automated processing, high sensitivity and long screening interval (5 years) [28]. It reminds us that if it can meet the requirements of the laboratory and personnel, screening with HPV test seems to be the preferred method for cervical cancer screening, which is consistent with the previous study conducted in China [29].

Another important result of this study is that there was no statistical difference in the total detection rate of CIN2+ between TS and LBC screening groups. Moreover, except for the eastern region, there was no statistical difference in the detection rate of CIN2+ between TS group and the other two groups in central and western regions. In recent years, because of its convenient sampling and high clarity, LBC has gradually replaced the traditional Pap smear as a cervical cancer screening method in the project counties of NCCSPRA. However, LBC needs not only special equipment and supplies but also trained specialists for interpretation. Unlike cytology, TS does not only examine surface epithelial cells. Light at specific frequencies is transmitted through cervical tissue identifying changes in the basal and stromal layers, including increases in blood circulation and variations in cell nuclei and cytoplasm that occur with precancerous change [9]. Moreover, compared with cytology and HPV, TS not only has the same advantages as HPV test: simple training and operation, automatic result generation, but also has the characteristics that real-time results can be obtained without the collection of cervical cell samples, or the help of laboratory equipment and cytologists [25]. Because of the characteristics of TS and the similar detection rate of CIN2+ to LBC, TS may become an effective screening test in areas where HPV test is nonexistent or unreliable.

In conclusion, in order to meet the global call for action towards the elimination of cervical cancer, in 2019, the State Council of China issued Healthy China Action (2019–2030), it specified cervical cancer screening targets for 2030: by 2030, cervical cancer screening for rural women should cover more than 90% of counties of China. For the presence of a large number of people to be screened, a lack of experienced cytopathologists and poor laboratory conditions make population-based screening program more challenging. The high detection rate of CIN2+, minimal training requirements, speed of analysis, automated features of HPV test make it ideal for primary cervical screening in regions where meet the requirements of the laboratory and personnel for HPV test. With similar detection effect to LBC and advantages such as being and objective and real-time test, makes TS an alternative way for cervical screening in low-resource regions where cytology screening cannot be effectively performed, that have a lack of HPV test conditions and have difficulties in patient follow-up, for example in central and western regions of China

### Strengths and limitations

There were several strengths to this study: Compared with previous studies, this study is the first large sample and population-based study on TS test. Except for HPV testing, all of the examination and test were performed by the grass-roots personnel who were involved in the NCCSPRA. It makes this study based on the real environment of cervical cancer screening. All of the participants of this study were screened by the three screening methods at the same time. It avoids the influence on the detection rate due to different screening populations and different incidence of cervical lesions. There are also some limitations to this study: In many NCCSPRA project counties where cytology test was used for primary screening, the follow-up rate of colposcopy was about 60% [30]. However, in this study, almost all the patients (96.8%) with abnormal screening results underwent colposcopy, which can weaken the advantage of TS that immediately available results can be obtained and if necessary to provide colposcopy at the same visit. China has a large population and unbalanced development. This study was only conducted in 8 counties in China and might not be representative of the rest of the country. However, despite these limitations, this study in the real world ensures the authenticity of the screening technology and the screening abilities of health personnel in different regions, and it can provide evidence for cervical cancer screening in China and the other developing countries and regions.
